# Establishing the psychometric properties of constructs from the conceptual ‘Settlement Services Literacy’ framework and their relationship with migrants’ acculturative stress in Australia

**DOI:** 10.1371/journal.pone.0266200

**Published:** 2022-04-05

**Authors:** Andre M. N. Renzaho, Michael J. Polonsky, Ahmed Ferdous, Adnan Yusuf, Julianne Abood, Bukola Oladunni Salami, Kerry Woodward, Julie Green

**Affiliations:** 1 Translational Health Research Institute, School of Medicine, Western Sydney University, Penrith, New South Wales, Australia; 2 Department of Marketing, Faculty of Business and Law, Deakin University, Burwood, Victoria, Australia; 3 Faculty of Nursing, Edmonton Clinic Health Academy, University of Alberta, Edmonton, Alberta, Canada; 4 Centre for Sustainable Communities, University of Canberra, Bruce, ACT, Australia; 5 Murdoch Children’s Research Institute, Parkville, Victoria, Australia; 6 School of Social Sciences, Western Sydney University, Penrith, New South Wales, Australia; 7 Department of Paediatrics, University of Melbourne, Parkville, Victoria, Australia; Universiti Pertahanan Nasional Malaysia, MALAYSIA

## Abstract

**Background:**

Effective migration often requires supports for new arrivals, referred to as settlement services. Settlement services literacy (SSL) is key to ensuring new migrants have the capability to access and utilise the information and services designed to support the resettlement process and achieve positive settlement outcomes. To date, however, no research has sought to empirically validate measures of SSL or to assess individual migrants’ levels of SSL. The aim of this study was to establish the psychometric properties of constructs from the conceptual SSL framework.

**Design:**

Using a snowball sampling approach, trained multilingual research assistants collected data on 653 participants. The total sample was randomly divided into two split-half samples: one for the exploratory factor analysis (EFA; N = 324) and the other for the confirmatory factor analysis (CFA; N = 329) and scale validation. The final SSL scale included 30 questions. The full data set was used to test the nomological validity of the scale regarding whether the components of SSL impact on migrants’ level of acculturative stress.

**Results:**

The EFA yielded five factors: knowledge (eight items, α = 0.88), empowerment (five items, α = 0.89), competence (four items, α = 0.86), community influence (four items, α = 0.82), and political (two items, α = 0.81). In the CFA, the initial model demonstrated a poor to marginal fit model. Its re-specification by examining modification indices resulted in a good model fit: CMIN/DF = 3.07, comparative fit index = 0.92, root mean square error of approximation = 0.08 and standardised root mean square residual = 0.07, which are consistent with recommendations. All the path coefficients between the second-order construct (SSL) and its five dimensions (knowledge, empowerment, competence, community influence and political) were significant at an α = .05 level, giving evidence for the validity of different SSL dimensions. We found that SSL is significantly related to migrants’ acculturative stress (β = - 0.39, p < 0.05) in the nomological model.

**Conclusions:**

The study provides evidence of the construct validity and reliability of the SSL tool. It provides the basis for integrating the measures of SSL into evaluation of settlement services. This will allow for more effective decision-making in designing and implementing settlement services as well as funding and service agreements to address any deficiencies.

## Introduction

The number of migrants accepted across Organisation for Economic Co-operation and Development (OECD) countries varies significantly. Some countries have relatively small numbers of migrants, such as Luxemburg, Israel, Finland or Denmark, whereas others attract significant numbers such as the United States of America, Germany or the United Kingdom [1]. Compared with other countries, Australia has received a large numbers of international migrants and is ranked sixth among OECD countries for the intake of permanent migrants and second for the proportion of its population born overseas(29.9%) [1]. Historically, net overseas migration has surpassed natural increases as the driver of the Australian population growth. For example, in 1977 natural increase and net immigration accounted for 67% and 33% of population growth respectively. By 2017, this trend had been reversed, with net immigration accounting for 64% of Australian population growth whilst natural increase contributed only 36% [2]. The contribution of net immigration to Australian population growth remained strong in 2020, accounting for 57% of population growth for the year to June 2020, which was against the backdrop of 12% decrease in new arrivals due to the COVID-19 pandemic [3].

Research demonstrates that migrants improve the performance and diversity of the labour markets (accounting for 47–70% of the workforce), pay more taxes and make more social contributions than they receive in benefits, and thus both have positive direct and indirect effects on economic growth [4, 5]. In Australia, based on the 2014–15 migrants intake, it has been projected that migrants will contribute a net fiscal benefit of $9.7 billion over the next 50 years [6]. Furthermore, there are broader social benefits of migration, such as enhanced multiculturalism, which is viewed as positive by 84% of Australians [7]. Integration of migrants in host societies, such as Australia, is therefore an essential priority. However, for migrants, navigating new country environments is challenging, especially when many of these migrants move to countries with significantly different cultural, economic, and social environments [5]. Transitioning to individualist cultures, such as in Australia, is especially difficult for migrants from collectivist cultures [5], who represent the majority of permanent migrants in Australia and more generally reflect most migrants from developing countries [8]. Of the top 10 countries of birth as at 30 June 2020 within Australia, six (India, China, Philippines, Vietnam, Malaysia, and Sri Lanka) are predominantly collectivist [9]. In collectivist cultures, there is a preference for an in-group identity where everyday life is characterised by group loyalty, respect for authority and hierarchical roles, interdependence, and lack of boundaries between private and work lives. In contrast, in many western countries, such as Australia, individualism is the norm, where personal choices, self-expression, independence, distinction between work and private lives are main characteristics of everyday life [10–13].

The clash between individualist and collectivist cultural orientation affects many aspects of migrant families including family functioning, parent-child relationships [14], and access to and utilisation of settlement services [15]. These social and cultural conflicts associated with adapting to a new culture are significant stressors which can be exacerbated by migrants’ language barriers, unfamiliarity with the host countries’ local context, unawareness of the supports that are available to assist with their settlement journey, and limited or no experience navigating complex service and information systems within their home countries [16–19]. Such challenges affect negatively multiple areas of migrants’ functioning, including changes in somatic, psychological and social wellbeing—changes collectively known as acculturative stress [20]. Therefore, accessing the various government supports to assist migrants overcome these challenges is essential.

In Australia, the Federal Government invests in substantial targeted services to assist migrants in their settlement journey. The migrant settlement service programs provided are diverse and encompass both the provision of specialised settlement services, as well as a broad range of social and human services provided specifically to new migrants. That is, in addition to government assistance (the pre-migration cultural orientation program) and access to mainstream social and health services provided by governments, community organisations and the private sector, migrants have access to specialised settlement services. [21] For example, the Adult Migrant English Program provides unlimited hours of English classes until migrants reach vocational English. The Humanitarian Settlement Program (HSP) helps new humanitarian arrivals build skills and knowledge needed to become self-reliant and active members of the community [22]. It incorporates the former Complex Case Support program[25], now known as Specialised and Intensive Services, which focus on migrants with complex or high needs not met by other settlement services. After being in the HSP for six to 18 months, eligible migrants and humanitarian entrants move to the Settlement Engagement and Transition Support (SETS) program, which provides long-term settlement services. The SETS program’s objective is to maximise migrants’ social participation, personal and economic well-being, independence, and community connectedness[23]. It incorporates tailored client services and community capacity building, and may refer clients to the AMEP and the Skills for Education and Employment program based on the clients’ needs. Then there is the free Translating and Interpreting Services for migrants, which help eligible individuals more generally overcome language barriers when accessing settlement and other social and community services [24].

Some scholars have argued that there is a mismatch between migration policies and post migration settlement outcomes, a situation that is exacerbated by receiving nations’ failure to recognise and address the interaction effects of global forces that directly affect international migratory pathways and associated settlement service needs [26, 27]. One of such factors is migrants’ ability to engage with host countries’ service delivery systems, such as a participatory approach [28], yet there have not been empirical measures of migrants’ ability to engage or providers’ assessment of service effectiveness [29]. The literacy lens is an approach that has been suggested that can be used to assess migrants’ abilities to engage with services designed to enhance inclusion in host communities [30]. The United Nations Educational, Scientific and Cultural Organization has provided a broad multidimensional definition of literacy applicable to the settlement context which is a “a continuum of learning in enabling individuals to achieve his or her goals, develop his or her knowledge and potential, and participate fully in community and wider society” [31] (p.13). Adoption of this broader definition of literacy lens has been used to explore other contexts as well, such as issues around more effective engagement with health services to improve health outcomes [32] as well as the use of technology [33]. However, the literacy lens for settlement services has never been empirically tested [30].

To date, no research has sought to empirically validate a measure of settlement services literacy (SSL) or to assess individual migrants’ levels of settlement service literacy. The identification of this is important for many reasons. At the migrant community level, this assessment will allow governments to identify areas where migrants are deficient and allow for more targeted services and interventions to be introduced, thereby enhancing the individual and community’s effective settlement inclusion. From a policy perspective, this also allows for an evaluation of the services provided by assessing SSL pre- and post-service experience and will allow service providers to better assess the effectiveness of their programs. This includes assessing whether individual programs are equally effective across targeted migrant groups. If effectiveness differences exist between groups, this would suggest adaption of programs is required to address specific community needs. Therefore, the aim of this study was to establish the psychometric properties of constructs from the conceptual ‘Settlement Services Literacy’ framework.

### The conceptual ‘Settlement Services Literacy’ scale

Literacy is defined in many, often contested, ways, with the most dominant construction stressing a technical, skill-based notion concerned with reading and writing [34]. A more extensive and socially situated definition accounts for the social and cultural factors that compete, shape and re-shape literacy [35] where literacy practices are always embedded in social life and cultural practices [36, 37]. This conceptualisation acknowledges the plural influences of everyday contexts of class, ethnicity, gender and generation [38] and other contextual community and individual factors such as the family, community, workplace, religious establishments, culture, history, language, and socio-economic conditions throughout an individual’s life [31]. These factors shape how literacy is acculturated into the flow of life and into the activities, processes and attitudes involved in meeting daily needs [39] highlighting interactive discourses of literacy and overall functioning and wellbeing [40]. This plurality also applies to SSL given the diversity of services available to new migrants. The concept of migrant SSL has been coined by Masinda [30] as a framework to conceptualise how new migrants develop abilities to know, understand, access, critically navigate and advocate for more effective settlement services. The framework refers to ‘settlement services’ to denote services that migrants receive pre- and post-migration to facilitate their settlement in a new country. Migrants’ access to and efficient use of such services is key to their successful integration in the new environment [30]. It has been suggested that new migrants with poor SSL lack the ability to access available information, services and supports provided to assist the resettlement process. For example, new migrants without established family, community and social networks in the host country are more vulnerable to social isolation, acculturative stress, and dependence on settlement services to provide culturally appropriate and relevant information fundamental to achieving positive settlement outcomes [41]. However, Masinda’s framework remains conceptual in nature and has not been validated to confirm its psychometric properties.

SSL encompasses the development of competencies to ensure new migrants have the skills and knowledge that enable them to interact effectively with settlement services, to access and use the information needed to contribute to their socio-economic development, reduce structural inequity, increase sense of belonging, promote successful settlement outcomes, and overall wellbeing. Masinda argues that the availability of migrant settlement services may not be sufficient itself if migrants lack the appropriate skills and aptitudes to fully utilise services available to them [30]. This may also explain variations in settlement service efficacy identified by providers [29].

Conceptually, Masinda suggested there are three levels of SSL, each with clearly defined indicators (Table 1) [30]. The first component, basic SSL, refers to new migrants’ foundational information, knowledge, and skills to access and effectively utilise settlement services. The second component, critical SSL, refers to new migrants’ ability to critically navigate and engage with settlement services. The third level, political SSL, refers to migrants’ skills and awareness to act as agents of change for settlement services. Recently, Abood, et al [42] undertook a systematic review of SSL and found that while this concept has not been extensively referred to in the literature, the three components were found to exist within the literature around settlement services. Yet, to date, no study has sought to establish the psychometric properties of the components of the SSL construct.

**Table 1:** Immigrant settlement services literacy indicators (adapted from Masinda 2014).

	Level of SSL	Indicators
**Immigrant settlement services literacy (ISSL)**	**Basic SSL** Individuals and communities have the skills enabling navigation, knowledge, and use of SS.	Read and understand migrant resource guides Know to use the internet to learn about migrant services Connected to friends from own and other communities Know the locations of SS Know the eligibility criterion of SS Know what to do to access SS
	**Critical SSL** Individuals and communities critically think about service quality, appropriateness, strengths, and gaps.	Capable to suggest potential solutions for better migrant services Critically think about service appropriateness, strengths, and gaps Familiar with migrants’ rights to appropriate services Capacity to question organisations about service planning, delivery, and evaluation
	**Political SSL** Individuals and communities are aware of their capacities to change the course of their integration.	Perceive community organisations as agents of change Ethnic communities’ awareness to influence policy about migrant services Ethnic community leaders are involved in political parties at different levels of government (federal, state, and local) Community leaders are represented on advisory and service committees Number of professionals in various domains of mutuality, civic, and community-based politics.

## Materials and methods

### Setting and target population

The study was carried out in New South Wales and Victoria, Australia; and was approved by the Western Sydney University Human Ethics Committee (HREC Approval Number: H13063). The study focused on migrants who have been in Australian for 5 years or less, living in local government areas with the highest proportion of migrants (where 37–56% of the population were born overseas), and an Index of Relative Socio-economic Disadvantage score of <1000, which is the cut-off used to indicate socio-economic disadvantage [43]. The five major language groups included in our study were Arabic, Burmese, Dari, Farsi, and Tamil, coming from a range of communities from different countries of birth.

The study also collected background information on each participant including, sex (female, male, other), age in years, pre-migration educational attainment (university or tertiary, technical or trade certificate, completed secondary school, some secondary school, completed primary school, some primary school, and never attended school), marital status (married, single, widowed, divorced, separated, or de facto), living structure (live with family, live with friends, live with family and friends, live alone, live alone but sometimes with others), employment status (self-employed, permanent /ongoing, fixed term contract, casual, and unemployed), years lived in Australia, and reason for migration (refugee/humanitarian entrant, family reunion, education opportunities, financial/economic, political/asylum seeker, and other reasons) (see Table 2).

**Table 2:** Demographic characteristics of the two split-half samples.

	*Total Sample*		*Sample 1*		*Sample 2*	
	N	Percent	N	Percent	N	Percent
	653	100	324	49.62	329	50.38
*Sex*						
Male	243	37.21	128	39.5	115	34.95
Female	409	62.63	196	60.5	213	64.74
Not revealed	1	0.20	0	0	1	0.30
*Age in years (mean ± SD)*	653	37.00 ± 11.82	324	37.37 ± 11.72	329	36.62 ± 11.91
*Pre-migration Education*						
University or tertiary	250	38.28	117	36.11	133	40.43
Technical or trade certificate	49	7.50	23	7.10	26	7.90
Completed secondary school	140	21.44	76	23.46	64	19.45
Some secondary school	87	13.32	42	12.96	45	13.68
Completed primary school	39	5.97	24	7.41	15	4.56
Some primary school	37	5.67	16	4.94	21	6.38
Other education	13	1.99	8	2.47	5	1.52
Never attended school	38	5.82	18	5.56	20	6.08
*Marital status*						
Married	445	68.15	228	70.37	217	65.96
Single	156	23.89	73	22.53	83	25.23
Widowed	26	3.98	10	3.09	16	4.86
Divorced	10	1.53	5	1.54	5	1.52
Separated	10	1.53	4	1.23	6	1.82
De facto	6	0.92	4	1.23	2	0.61
*Living structure*						
With family	595	91.12	297	91.67	298	90.58
With friends	24	3.68	10	3.09	14	4.26
With family and friends	13	1.99	7	2.16	6	1.82
Alone	12	1.84	5	1.54	7	2.13
Alone but sometimes with others	1	0.15	0	0.00	1	0.30
No response	8	1.23	5	1.54	3	0.91
*Employment status*						
Self employed	4	0.61	2	0.62	2	0.61
Permanent /ongoing	104	15.93	54	16.67	50	15.20
Fixed term contract	26	3.98	15	4.63	11	3.34
Casual	69	10.57	30	9.26	39	11.85
Other	7	1.07	3	0.93	4	1.22
Unemployed	443	67.84	220	67.90	223	67.78
*Years in Australia (mean ± SD)*	653	2.64 ± 1.34	324	2.57 ± 1.32	329	2.70 ± 1.35
*Migration type*						
Refugee/Humanitarian Entrant	397	60.80	198	61.11	199	60.49
Family reunion	98	15.01	55	16.98	43	13.07
Education opportunities	56	8.58	26	8.02	30	9.12
Financial/economic	54	8.27	21	6.48	33	10.03
Political/Asylum Seeker	14	2.14	10	3.09	4	1.22
Other reasons	34	5.21	14	4.32	20	6.08
Country/Region of Origin						
Iraq	60	18.5	48	14.6	108	16.5
Syria	59	18.2	56	17.0	115	17.6
Afghanistan	47	14.5	59	17.9	106	16.2
Sub-Saharan Africa	44	13.6	38	11.6	82	12.6
Iran	39	12	26	7.9	65	10.0
Myanmar	25	7.7	37	11.2	62	9.5
Bangladesh	20	6.2	20	6.1	40	6.1
Nepal	15	4.6	25	7.6	40	6.1
India	15	4.6	20	6.1	35	5.4

### Study design, sampling strategy

After receiving ethical institutional clearance, a cross-sectional survey was carried out between 7 August– 9 December 2020. Multilingual research assistants representing the target communities were recruited and trained to implement the survey and ensure appropriate duty of care towards respondents, as well as maintaining data quality and integrity. Recruiting multilingual research assistants from the target communities ensured that materials and research questions could be delivered in a language relevant to the various communities, as well as to ensure we were working with various communities, rather than simply researching these communities [44]. The participant information sheet, consent form, and revocation of consent form were translated into the five community languages used in our study—English as well as Arabic, Burmese, Dari, Farsi, and Tamil. Using a snowball sampling approach, multilingual research assistants recruited members of their respective communities in the study. Participants were recruited through our multilingual research assistants’ contacts, community associations, social media (Facebook), referrals from local migrant services, and via ethno-specific and religious community groups. Acquaintances in turn referred their friends and/or family to the research team. This sampling technique is considered best for difficult-to-access populations [45], such as newly arrived migrants experiencing language barriers, culture shock, and always on the move in search for affordable housing [10, 11, 46]. We attempted to limit potential bias and improve representativeness by focusing the sampling in local government areas with the highest concentration of the target migrant communities to ensure inclusiveness and adequate coverage of the target population. In each local government area, the multilingual research assistants and research team members distributed leaflets and posters throughout multilingual research assistants’ networks, community associations, migrant service providers, religious institutions, and community facilities. All materials were available in English, Arabic, Burmese, Dari, Farsi, and Tamil. We sought to have representative sample quotas and the community mobilisation continued until each quota was filled or each multilingual research assistant was unable to recruit further participants in their community group and local government area. The study adhered to the Strengthening the Reporting of Observational Studies in Epidemiology (STROBE) guidelines (S1 Appendix).

### Data collection and sample size

The survey instruments were administered using either face-to-face or online (phone and via video platforms such as Zoom and Skype) approaches, as the data collection was undertaken before, during, and after various COVID 19 lockdowns in NSW and Victoria. The interviews were conducted in participants’ preferred language and preferred location including homes, public libraries, community organisations, public parks, and cafes. Respondents were compensated with a $20 gift voucher for their time and effort, as the survey took over 90 minutes to complete. In total, 792 respondents participated in the study, however only 679 participants provided usable data, giving a usable sample of 85.7%. Of the usable sample, a further 26 participants provided incomplete data on the sub-scales of interest, hence excluded from the final analysis, giving us a total sample size of 653 (missing data-adjusted participation rate of 82.4%). With our final scale containing 30 items, the sample required for our construct validity and reliability analyses was 10 participants per item (~300 participants) for stable factor analyses [47]. The sample size was doubled to allow us to systematically assess the scale, using a split sample approach whereby we could undertake psychometric testing of the scale.

### Training multilingual research assistants

Data were collected by 39 multilingual research assistants (17 in NSW and 22 in Victoria), who were trained and supervised by two project officers experienced in data collection monitoring and data quality assurance and control. Multilingual research assistants received three hours of training followed by a dry run prior to data collection to ensure they were familiar with the study instrument and instrument administration procedures. The training covered sampling techniques and how to minimise biased oversampling, interview techniques, and ethical issues including confidentiality, and professional conduct, as well as COVID safe practices, in line with university requirements.

### Measures

Masinda’s initial conceptual SSL scale included 30 questions, 10 per domain (see S1 Appendix). A four-step validation process was undertaken: (1) The items proposed by Masinda were assessed to ensure their face validity (suitability, relevance, and clarity); (2) the data were then collected, with the sample split randomly, where the first group was used to undertake exploratory construct validity and reliability analysis; (3) the second half of the sample was then examined using confirmatory construct validity; and (4) the two sets were then pooled and the nomological validity of the scale was assessed. This multi-stage approach is often used in scale development across a range of disciplines [48, 49]. The first step involved the items being reviewed by the research team to establish their face validity. The review of the scale was complemented by a systematic review of the SSL literature to ensure that the items covered the comprehensive range of issues identified within settlement service the literature related to SSL [42]. This process resulted in all 30 original items being retained, with four items revised to improve their clarity.

### Migrants’ level of acculturative stress

To assess nomological validity we evaluated whether the SSL reduced migrants’ level of acculturative stress, as measured by the Social, Attitudinal, Familial, and Environmental Acculturative Stress (SAFE) Scale [50]. Extensive research suggests that migrants who are better able to engage with settlement services have lower levels of acculturative stress [51]. As such, nomological validity would occur when increased SSL reduces migrants’ level of acculturative stress [42]. We assessed the migrant’s level of acculturative stress using the 13item SAFE Scale which was previously validated by Suh, Rice (50).

### Statistical analysis

The sample was randomly divided into two split-half samples [52]. The first half was used for the exploratory factor analysis (EFA; N = 324) to determine the factor structures and the second half was used for the confirmatory factor analysis (CFA; N = 329) to provide the evidence for the factor structures as summarised in Table 3. We then used the full data set to test the nomological validity of the scale regarding whether increases in the components of SSL reduced migrants’ level of acculturative stress. The EFA was undertaken using maximum likelihood method with oblique rotation to determine the factor structure. The number of factors retained was determined by the Scree test (i.e., a discourteous break in eigen values), and factors also had to have an Eigen value greater than 1 [52]. A factor was retained if it: had at least three items with significant loadings >0.5 and 2) the items and the factor shared some conceptual meaning. Any items that cross-load on another factor (cross-loading > 0.4), were also removed. The Kaiser–Meyer–Olkin (KMO) index was used to measure the homogeneity of variables and to check if the data were suited for factor analysis, with KMO >0.60 used as a cut-off point to confirm the suitability of factor analyses [52]. Cronbach alpha (α) was used to assess the internal consistency reliability of the scales as follows: α≥0.9 = excellent, 0.7≤α< 0.9 = good, 0.6≤α<0.7 = acceptable, 0.5≤α≤0.6 = poor, and α<0.5 = unacceptable.

**Table 3:** Factor loadings, item means, standard deviation (SD), and Cronbach alpha coefficients for the two samples.

Factor & Items	Item description	Sample 1: *N = 324*			Sample 2: *N = 329*		
		EFA Loading	Mean	SD	CFA Loading	Mean	SD
Knowledge (α = 0.88)							
KNOW1	I know about SS and where they are located	0.77	3.11	1.13	0.65	3.14	1.23
KNOW2	I know the location of specific programs for immigrants	0.77	2.94	1.19	0.72	3.00	1.22
KNOW3	I know the eligibility criteria for various SS	0.63	2.74	1.19	0.78	2.74	1.26
KNOW4	I know where to find culturally sensitive SS	0.70	2.63	1.13	0.75	2.73	1.20
KNOW5	I know which SS are or are not relevant to me	0.66	2.92	1.17	0.81	2.88	1.28
KNOW6	I have a sense that SS providers listen to me	0.67	3.40	0.97	NA*	NA	NA
KNOW7	I know how to seek help with SS	0.76	3.32	1.07	NA	NA	NA
KNOW8	I communicate to SS providers	0.77	3.24	1.15	NA	NA	NA
Empowerment (α = 0.91)							
EMP1	I can change the course of the way SS are provided	0.77	2.30	1.06	0.89	2.28	1.11
EMP2	I can influence how programs around SS are initiated	0.81	2.35	1.12	0.88	2.32	1.13
EMP3	I can change policy around SS issues	0.83	2.13	1.07	0.87	2.07	1.14
EMP4	I can influence policy around SS	0.85	2.23	1.06	0.91	2.19	1.18
Competence (α = 0.86)							
COMP1	I read and understand immigrant resources guides	0.70	3.06	1.25	0.71	3.12	1.22
COMP2	I use the internet	0.81	4.10	1.16	0.73	4.17	1.22
COMP3	I can use the internet to look for SS	0.83	3.40	1.39	0.88	3.57	1.38
COMP4	I can read and understand printed SS material	0.83	3.31	1.36	0.88	3.48	1.39
Community Influence (α = 0.82)							
CINF1	Community leaders participate in political parties	0.81	2.89	0.96	0.76	2.91	1.09
CINF2	Ethnic community leaders are aware of their strengths	0.74	3.14	0.92	0.72	3.16	1.06
CINF3	Ethnic community leaders have political skills	0.84	2.98	0.94	0.86	2.98	1.03
CINF4	Community professionals are involved in political mobilisation	0.72	2.88	0.95	0.85	2.90	1.05
Political participation (α = 0.81)							
POL1	I am involved in political parties that support SS	0.83	1.69	0.94	0.80	1.48	0.87
POL2	I am involved in politics to change SS policy	0.85	1.60	0.95	0.81	1.43	0.83

EFA = Exploratory factor analysis; CFA = Confirmatory factor analysis; SS = Settlement services

*NA: These items were not included in the final CFA model.

The CFA used the maximum likelihood estimation to validate the factor structure. The following indices and associated cut-off points were used to evaluate the model and to indicate a good fit: the chi-square associated with each degree of freedom (CMIN/DF) < 3, Comparative Fit Index (CFI) ≥ 0.90, Root Mean Square Error of Approximation (RMSEA) ≤ 0.08 and Standardised Root Mean Square Residual (SRMR) ≤ 0.08 [53].

To test for nomological validity we examined whether the hypothesised construct of SSL is significantly related to migrant’s level of acculturative stress in a nomological network. While a number of covariates have been found to impact on migrants’ acculturative stress [54], given we are focusing on support services for new migrants, we included length of stay in Australia, as those who have been here longer will have had a greater opportunity to engage with services and the type of migrant status, as some programs are targeted at different categories of migrants. However, an extended model that also included additional demographic characteristics of age, sex, and marital status as co-variates in the nomological network was produced with relationships consistent across the two models (see S1 Appendix for the expanded model).

As we collected cross-sectional data, where all variables were from individuals at one point in time, common method bias potentially poses a threat to our study’s results. To test the common method variance (CMV) in our nomological model, we controlled for the effects of an unmeasured latent factor using Harman’s single-factor approach [55].

## Results

The demographic characteristics of participants are summarised in Table 2. The mean (standard deviation) age and length of stay in Australia was respectively 37.0 (11.8) and 2.6 (1.3) years. Almost two thirds of the sample (62.6%) were females, whilst 67.2% completed secondary school or higher prior to migration. Our study participants were predominantly married (68.2%), living with family members (91.1%), unemployed (67.8%), and refugees or humanitarian entrants (60.8%).

The Kaiser-Meyer-Olkin (KMO) coefficient was 0.85 and Bartlett’s Sphericity test was found to be significant *χ*2 (df = 276), = 4364.59, p < 0.001), indicating factor analyses were justified. However, 8 items of the 30 SSL items did not load sufficiently on any one factor (loading < 0.5) or had high cross-loadings (loading ≥ 0.4) on other factors: ‘My income allows me to make it to the end of the month’, ‘My culture is distant from that of Australians’, ‘I use the instructions given to me by SS providers’, ‘I am aware of the gaps in SS’, ‘I’m thinking about getting involved in politics to change SS policy’, ‘My community can change SS policy’, ‘My community is organised as an agent of change (ie. they can create change in society)’, and ‘I have the capacity to question SS agencies’. These items were dropped and the remaining 22 items retained the five factors, Each of the 22 items loaded satisfactorily onto one of the factors (factor loadings > 0.5) without high cross-loadings (≥ 0.4) [56]. The final five factors of the EFA model were: knowledge (eight items, α = 0.88), empowerment (four items, α = 0.91), competence (four items, α = 0.86), community influence (four items, α = 0.82), and political participation (two items, α = 0.81), although two item measures are potentially an issue [56]. Eigen values were found to be: 7.05 for knowledge, 3.25 for empowerment, 2.40 for competence, 2.02 for community influence and 1.25 for political participation. The extraction of the five factors accounted for 66.5% of the total variance in the data (Table 4).

**Table 4:** Establishing discriminant validity of SSL dimensions.

	SSL Dimension	CR	AVE	MSV	1	2	3	4	5
1	Knowledge	0.86	0.55	0.25	**0.74**				
2	Empowerment	0.89	0.80	0.25	0.40***	**0.89**			
3	Community Influence	0.88	0.64	0.10	0.22***	0.32***	**0.80**		
4	Competence	0.88	0.64	0.25	0.50***	0.50***	0.29***	**0.80**	
5	Political participation	0.79	0.66	0.18	0.19**	0.42***	0.19**	0.08	**0.81**

SSL = Settlement Services Literacy

CR = Critical Ratio

AVE = Average Variance Extracted

MSV = Maximum Shared Variance (squared correlations between dimensions)

Values in normal font represents correlations among SSL dimensions, and values in bold font represent the average factor loadings for items of each SSL dimension.

**Fig 1:**
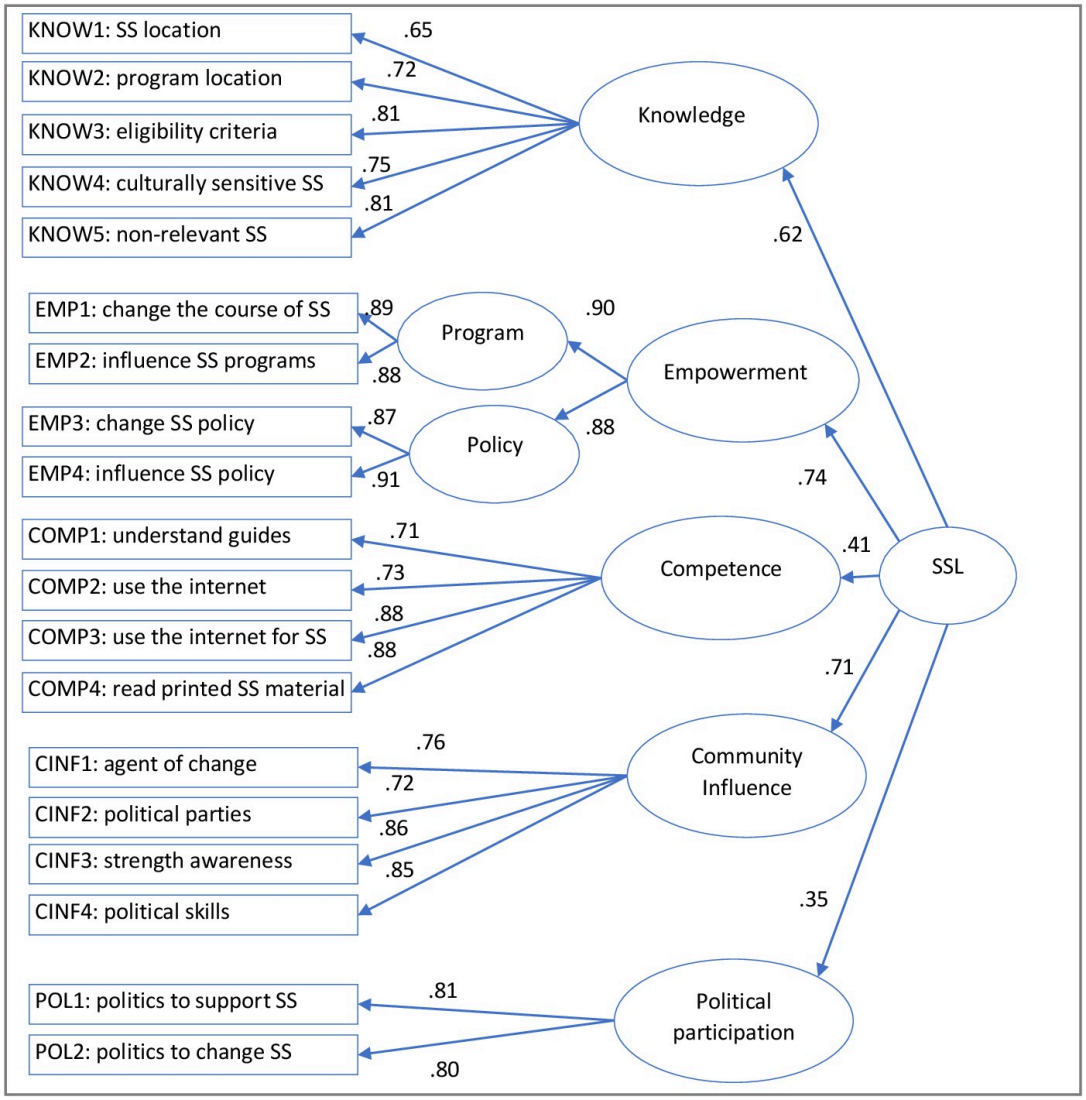
Hierarchical model for SSL for CFA. SS = Settlement services.

In the CFA, the findings of the second-order SSL construct based on the 5 dimensions and 22-item scale suggest that the initial model demonstrated a poor to marginal fit model (see Fig 1). Modification indices obtained from the initial tests of the model suggested that the model fit would improve with additional item deletion. Based on high values of modification indices we iteratively deleted three additional items from our CFA analysis: KNOW6 (I have a sense that settlement services’ providers listen to me), KNOW7 (I know how to seek help with settlement services), and KNOW8 (I communicate to settlement services’ providers). The final model in summarised in Fig 1. Before deleting any item from the model, we ensured that the domain of the construct did not change. Similarly, from a domain sampling viewpoint, only the best set of indicators that are closer to the centroid of a reflective construct should be included in the model. Because SSL is hypothesised to be a higher-order reflective construct with reflective first-order dimensions, as well as reflective indicators for each dimension, item deletion did not pose a threat to the validity of the construct to the extent we have sufficient indicators to measure the construct. Modification indices suggested that the model fit would improve if we allowed the errors between EMP1 (I can change the course of the way settlement services are provided) and EMP2 (I can influence how programs around settlement services are initiated) to covary. This modification is theoretically grounded because these two items relate to the perceived empowerment to influence or change how settlement services are delivered. The other two items related to the empowerment dimension, EMP4 (I have the capacity to question settlement services agencies) and EMP5 (I can change policy around settlement services’ issues), relate to the perceived empowerment to influence settlement services-related policies. Therefore, we respecified our measurement model by introducing the program and policy related sub-dimensions to the first-order empowerment dimension (Fig 1).

The modification in the model resulted in a good model fit based on the accepted fit indexes: CMIN/DF = 3.07, CFI = 0.92, RMSEA = 0.08 and SRMR = 0.07, which are consistent with requirements for acceptable model fit [53]. The path coefficients between the indicators and their respective first-order factors were significant at an α = .05 level, and the standardised loading of each indicator to its hypothesised dimension is greater than 0.70 (except for 1 indicator: KNOW1, which has a factor loading of 0.66 on the knowledge dimension). This result gives satisfactory evidence for individual indicator validity. The Average Variance Extracted (AVE) for each SSL dimension is > 0.5 (see Table 5), which establishes the reliability of the set of indicators for each dimension. In addition, all the path coefficients between the second-order construct (SSL) and its five dimensions (knowledge, empowerment, competence, community influence and political) were significant at an α = .05 level (see Table 4), giving evidence for the validity of different SSL dimensions. The shared variance between each set of SSL dimensions is less than 0.5, and the maximum shared variance of each SSL dimension with other SSL dimensions is always lower than the AVE of each SSL dimension, providing evidence for discriminant validity of SSL dimensions (see Table 4).

**Table 5:** Dimensions of settlement service literacy.

Masinda conceptual dimensions	Empirical Results of this study
**Basic SSL** Individuals and communities have the skills enabling navigation, knowledge, and use of SS.	**Knowledge**- knowing what is available and where to access settlement services.
	**Competence**- being able to effectively engage with settlement service systems.
**Critical ISSL** Individuals and communities critically think about service quality, appropriateness, strengths, and gaps.	**No dimension identified**
**Political ISSL** Individuals and communities are aware of their capacities to change the course of their integration.	**Community Influence**- migrants have the ability to influence settlement services’ processes and activities
	**Empowerment**- migrants have the ability to shape policy as well as the programs delivered
	**Political**- migrants could use political processes to change actions as well as directions of government

**Fig 2:**
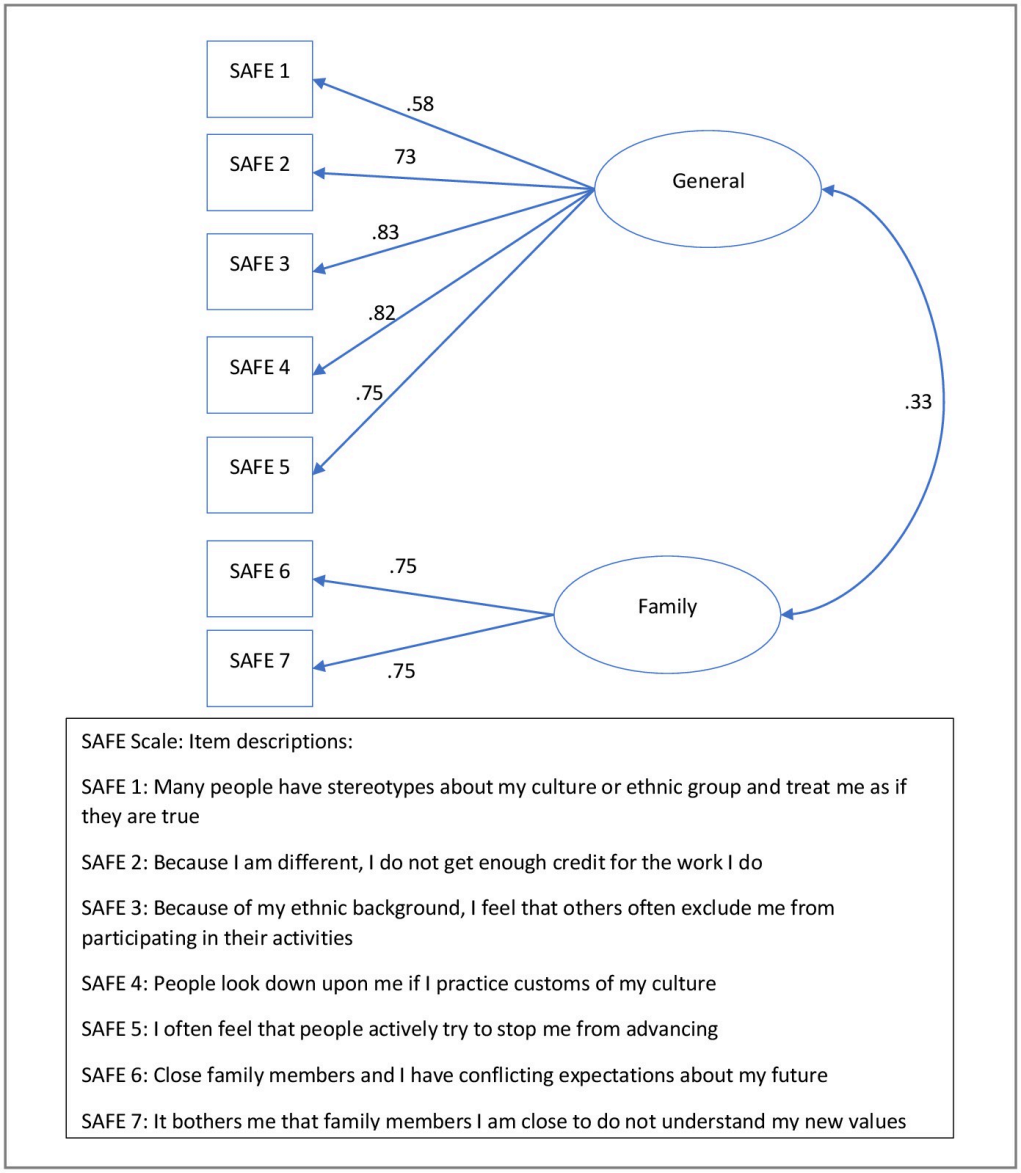
CFA analysis for SAFE.

Whereas, some researchers have assessed stress as a single dimension construct [57], in this study the migrant’s level of acculturative stress was measured using the validated 13 SAFE Scale [50]. The validated SAFE scale has two dimensions–(1) General stress with 10 items, and (2) Family stress with 3 items. We undertook a CFA to confirm the dimensionally of the scale. The CFA identified that 5 of the original items related to the General dimension and 1 item related to the family dimension did not appropriately load (SMC <0.3) on to the scale, and thus these were omitted. The final SAFE scale included 5 general stress items and 2 family stress items. This model resulted in a good model fit based on the accepted fit indexes: CMIN/DF = 1.26, CFI = 0.99, RMSEA = 0.03 and SRMR = 0.03, which are consistent with requirements for acceptable model fit [53]. The path coefficients between the indicators and their respective first-order factors were significant at an α = .05 level, and the standardised loading of each indicator to its hypothesised dimension was greater than 0.70 (except for 1 indicator: SAFE1, which has a factor loading of 0.58 on the General dimension). The loading of the individual items on the two sub-dimensions are provided in Fig 2.

**Fig 3:**
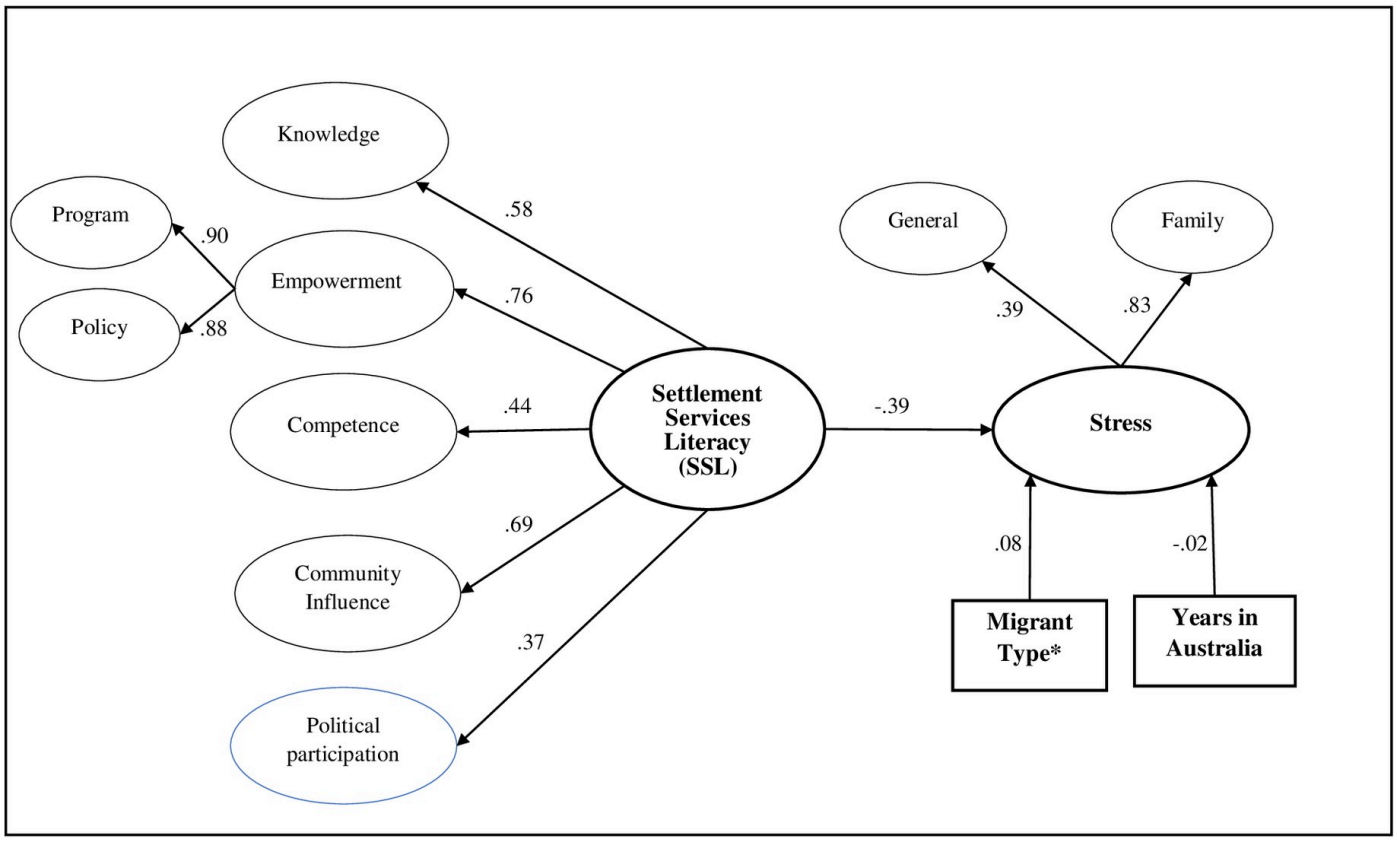
Establishing nomological validity for SSL. *Migrant type is measured as migrants who immigrated into Australia on refugee/humanitarian visa (coded as 1) versus all other migrant types as the reference group.

In testing the nomological validity, the results show that SSL is significantly related to Acculturative stress (β = - 0.18, p < 0.05). Fig 3 presents the nomological model and includes the estimates of relationships between constructs. Table 6 includes results of the nomological analysis including length of stay in Australia and type of migrant status as covariates. An extended nomological network model that included additional demographic characteristics of age, sex, and marital status as co-variates (See S1 Table). Results in the extended model show that the negative association between SSL and acculturative stress remained significant at 95% confidence interval. To test the CMV in our nomological model, we controlled for the effects of an unmeasured latent factor in our seven-factor model using Harman’s single-factor approach [55]. In this procedure, all the items were allowed to load on their respective theoretical constructs, as well as on a latent common method variance factor. We found that all the items loaded significantly on their theoretical constructs in the presence of the latent common method variance factor in our model. Further, the *χ*2 difference between the hypothesized model (*χ*2 (df = 271) = 610.934) and the constrained model (*χ*2 (df = 270) = 610.037) was non-significant (Δ*χ*2(df = 1) = 0.897, P > 0.10). Hence, common method bias did not appear to pose a serious threat to the study’s results.

**Table 6:** Results of the nomological validity analysis.

Dimension/Construct	Direction	Construct	Std. loading	Unstd. loading	Std. error	t-value	p-value
Acculturated stress	←	SSL	-0.39	-0.18	0.08	-2.19	0.03
Acculturated stress	←	Years in Australia	-0.02	0.00	0.01	-0.21	0.84
Acculturated stress	←	Migrant type *	0.08	0.04	0.04	0.98	0.33
General	←	Acculturated stress	0.39	2.50	1.11	2.25	0.02
Family ^#^	←	Acculturated stress	0.83	1.00	-	-	-
Knowledge ^#^	←	SSL	0.58	1.00	-	-	-
Empowerment	←	SSL	0.76	1.31	0.21	6.42	< 0.01
Competence	←	SSL	0.69	1.19	0.19	6.33	< 0.01
Community Influence	←	SSL	0.44	0.65	0.13	5.07	< 0.01
Political participation	←	SSL	0.37	0.48	0.12	4.10	< 0.01
Program ^#^	←	Empowerment	0.90	1.00	-	-	-
Policy	←	Empowerment	0.88	0.98	0.09	10.46	< 0.01

* Migrant type is measured as migrants who immigrated into Australia on refugee/humanitarian visa (coded as 1) versus all other migrant types as the reference group.

^#^ Only standardized loadings are reported for these relationships because path coefficients were fixed to 1 for scaling purposes. All these relationships were significant at 95% confidence interval.

## Discussion

The aim of the study was to establish the psychometric properties of constructs from the conceptual SSL framework. Our findings support a good model fit with strong construct and nomological validity. While we based our scale on Masinda’s work, our findings do not support Masinda’s three conceptual component [30]: basic, critical, and political factors. Using the proposed items resulted in in identifying five indicators that relate to settlement services’ literacy: knowledge, empowerment, community influence, competence, and political factors. Rather than find a single basic level of SSL, these appeared to be linked to two separate dimensions reflecting knowledge of the system and individuals being able to effectively engage with the system (competence). We did not identify what Masinda [30] referred to as a critical stage, but rather identified three subcomponents of the political stage. There was a component focused on empowerment, where migrants identified that they had the ability to shape policy as well as the programs delivered. There was also a recognition of the ability of the migrant community to influence processes and activities, referred to Community Influence. Finally, there was a more traditional political dimension, whereby migrants saw that they could use political processes to change actions as well as directions of government. As such, the empirical model appears to identify the complexity of the various components, but also appeared to really only cover two of the three dimensions proposed by Masinda [30]. Our new factors are supported by our nomological testing, which suggests a significant relationship between our identified dimensions and migrants’ level of acculturative stress. Our findings are consistent with the literature suggesting that high levels of acculturative stress due to issues associated with navigating the resettlement service delivery system [42, 58]. Migrants who are poorly informed of rights or disempowered, and lack an understanding of settlement service delivery options have been found to have high level of acculturative stress [42, 58].

Service literacy issues, particularly language skills, make it difficult for some new migrants to access settlement services [42]. For example, over 200 languages are spoken in homes and communities all over Australia. When engaging with settle services migrants are faced with a broad spectrum of providers delivering many of the same services. As such, the complexity of the service landscape can further limit service utilisation [16, 59]. For instance, there are multitude of organisations implementing settlement services, competing for the same funding sources, hence creating competing interests, though attention to collaboration within services is growing [59, 60]. This makes coordination and integration of settlement services a challenge. Migrants therefore must choose from a complex menu of existing settlement services, for which they may have varying degrees of understanding what programs are provided and appropriate for their needs. Moreover, migrants have little input into settlement services available to them nor are available services based on objective assessment and prioritisation of their needs.

Previously there was no way to empirically assess migrants’ level of SSL and as such the validated SSL scale has implications for both policy and services. The tool can be used to identify specific migrant or cultural groups that may face particular challenges in knowing about, accessing and utilising settlement services. It can also be used to assist settlement service providers to identify other literacy related barriers their clients face and to adapt services as required. It would also be possible to use the tool to evaluate the degree to which individual programs overcome gaps in migrants’ SSL. Although various programs may be designed to achieve differing objectives and thus any one program is unlikely to deliver on all dimensions of SSL. Understanding the components of SSL that provide migrants with the agency to influence the vision and delivery pathways of settlement services is timely and should form the basis for needs-based response design and intervention choices. Doing so may also facilitate inter-organisation coordination and planning to alleviate the acculturative stress associated with navigating settlement services. For example, there is extensive overlap between settlement services funded by the federal and state governments, and services are often accountable to funding agencies and not the beneficiaries. This funding model may reflect service duplication, however it could also reflect more targeted sub-programs aimed as migrant groups with specific needs. The tool can help to identify specific areas of SSL that migrants or specific migrant groups might need assistance developing. For instance, additional assessment could evaluate whether migrants require knowledge about what settlement services are available to them, or competency related issues regarding accessing information about how to utilise available services. Such knowledge can help target policy initiatives, as well as enhance specific service delivery. This would hopefully result in directing resources more effectively to maximise service effectiveness.

The study is subject to some limitations. Notably, while the items were assessed for content validity, there may have been additional qualitative validation prior to these being used in the quantitative survey that could have yielded more meaningful items for the tool. The fact that we did not identify a component assessing the critical SSL is something that needs further exploration. It may be that this is in fact more integrated into the other dimensions and thus is not assessable as a distinctive dimension of SSL. Of course, it could be that the items proposed by Masinda [30] did not adequately capture this domain, especially domains related to citizen power, individual and community agency, proactivity, and migrant associations for rights activation to question the quality of settlement services and propose solutions for better migrant settlement services. The tool was developed and tested in one country and while we included multiple migrant groups, it does require further exploration in other international settings. The use of multiple groups of migrants does suggest that the scales apply within the Australian context. However, the survey tool was translated into five community languages plus the English version for participants who preferred to be interviewed in English (e.g. those from English speaking countries such as Indian migrants). Having multiple languages reduced the cell sizes and limited our ability to measure and test invariance. Measuring invariance or measurement equivalence to assess whether SSL is being measured across the various languages was not the main objective of the study and constitutes a separate study in its own right. The political participation sub-scale had two items. While some literature suggests that factors should have three or more items, some authors have found two item constructs to be acceptable and a starting point for the potential development of a more complete subscale [61–63]. Therefore, we believe that deleting the political dimension would reduce the coverage of the SSL domain, which would be problematic. It may be possible to expand the items used for the political dimension in further SSL construct testing. Finally, the context examined was Australia and other countries have very different settlement service supports, as such further research is needed to explore other types of support structures.

## Conclusions

Settlement service literacy is key to ensuring new migrants’ have the capability to access and utilise the information and services designed to support the resettlement process and achieve positive settlement outcomes. However, little attention has been given to the level of migrant’s settlement service literacy. This study provides evidence of the construct validity and reliability of the SSL tool. The tool can provide a basis for integrating SSL into decision-making in settlement services’ design and implementation, as well as funding and service agreements.

## Supporting information

S1 Appendix STROBE checklist. (DOCX)

S1 Table Results of the extended nomological validity analysis with additional demographic variables of age, sex and marital status as co-variates. (DOCX)

S1 Dataset (TXT)
