# Supplementary material for: Establishing the psychometric properties of constructs from the conceptual ‘Settlement Services Literacy’ framework and their relationship with migrants’ acculturative stress in Australia
Source: PLoS One. 2022 Apr 5;17(4):e0266200. doi: 10.1371/journal.pone.0266200 (PMC8982835; doi:10.1371/journal.pone.0266200)
Supplement: S1 Table — (DOCX) [file pone.0266200.s002.docx]

| **Supplementary Table 1: Results of the Extended Nomological Validity Analysis with Additional Demographic Variables of Age, Sex and Marital Status as Co-variates** | | | | | | | |
| --- | --- | --- | --- | --- | --- | --- | --- |
| Dimension/Construct | Direction | Construct | Std. loading | Unstd. Loading | Std. error | t-value | p-value |
| Acculturated stress | 🡨 | SSL | -0.30 | -0.15 | 0.07 | -2.08 | 0.04 |
| Acculturated stress | 🡨 | Years in Australia | -0.03 | -0.01 | 0.02 | -0.36 | 0.72 |
| Acculturated stress | 🡨 | Migrant type ^1^ | 0.12 | 0.06 | 0.05 | 1.28 | 0.20 |
| Acculturated stress | 🡨 | Age | 0.06 | 0.00 | 0.00 | 0.61 | 0.54 |
| Acculturated stress | 🡨 | Sex ^2^ | 0.13 | 0.06 | 0.04 | 1.45 | 0.15 |
| Acculturated stress | 🡨 | Marital status ^2^ | 0.15 | 0.08 | 0.05 | 1.64 | 0.10 |
| General^#^ | 🡨 | Acculturated stress | 0.40 | 1.00 | - | - | - |
| Family | 🡨 | Acculturated stress | 0.81 | 1.79 | 0.75 | 2.40 | 0.02 |
| Knowledge^#^ | 🡨 | SSL | 0.57 | 1.00 | - | - | - |
| Empowerment | 🡨 | SSL | 0.68 | 1.26 | 0.19 | 6.47 | < 0.01 |
| Competence | 🡨 | SSL | 0.81 | 1.45 | 0.22 | 6.60 | < 0.01 |
| Community Influence | 🡨 | SSL | 0.40 | 0.60 | 0.12 | 4.80 | < 0.01 |
| Political | 🡨 | SSL | 0.28 | 0.40 | 0.11 | 3.53 | < 0.01 |
| Program^#^ | 🡨 | Empowerment | 0.93 | 1.00 | - | - | - |
| Policy | 🡨 | Empowerment | 0.86 | 0.93 | 0.09 | 10.09 | < 0.01 |

^1^ Migrant type is measured as migrants who immigrated into Australia on refugee/humanitarian visa versus all other migrant types as the reference group.; ^2^ Sex compares females with males as the reference group; ^3^ Marital status is measured as respondents who are married/in a de facto relationship, versus all others as the reference group as a reference group.

# Only standardized loadings are reported for these relationships because path coefficients were fixed to 1 for scaling purposes. All these relationships were significant at 95% confidence interval.
